# Institutional Notes and News

**Published:** 1909-12-04

**Authors:** 


### Institutional Notes and News.

### THE MIDDLESEX HOSPITAL AND CANCER RESEARCH.

The Court of Governors of Middlesex Hospital was held on November 25, with Major-General Lord Cheylesmore in the chair, in place of Prince Francis of Teck who was prevented from attending. The reception of the report was agreed to, and among the noteworthy donations recorded therein was King Edward's subscription of £100. Mention was also made of the quarter of a million left by Mr. Harry Barnato for the purpose of founding some charity in the nature of a hospital. A cancer institution in relationship to the Middlesex Hospital has been proposed.

### COVENTRY HOSPITAL.

At the last general meeting of the Coventry Hospital it was announced that the reorganisation sub-committee had made the following recommendations, and that these had been approved by the House Committee : (1) That a card index system for the registration of both in- and out-patients be adopted. (2) That in future the House Committee should meet not less than twice a month and that the laws be altered accordingly. (3) That the cost of endowing a bed be reduced to £500, and that the laws be altered accordingly, and that, as there is no provision in the laws for endowing a cot, it is recommended that it be added to the laws that a cot be endowed for £250. (4) That fire drills for the staff be organised, and that they be held once a fortnight, the Senior House Surgeon to act as senior officer, and that rules be drawn up by the House Surgeon and Secretary and submitted to the Committee at a future meeting.

### TORQUAY HOSPITAL.

The Western Hospital, Torquay, which is one of the oldest consumption hospitals in the Kingdom, has been improved and extended by the addition of two new wings. The administrative wing has an entrance porch and hall, both much larger than those superseded, with floors of terrazzo. Opening upon the hall are a dispensary, male and female isolation wards, nurses' and matron's sitting rooms, and a Board-room. The other wing has on each of its two floors bath-room, lavatory, and w.c. accommodation—for men on the ground floor and for women on the first floor. A new hot-water system of copper throughout and independent of the old supply has been installed to supply the new baths, lavatory basins, etc. The whole of the old drains have been removed, and a new system has been laid in the most approved manner, and provided with manholes of white glazed brick for the purpose of inspection and access to all branches. The new portions of the building are lighted by electricity, extended from the old premises. The alterations and extensions have been efficiently executed by Messrs. J. C. Parker and Sons, Union Street, to the plans and specifications of Messrs. Waymouth, Johnson and Webber, of the Tormoham Manor Office.

### SALFORD ROYAL HOSPITAL.

Sir Lees Knowles presided at the annual meeting of the Salford Royal Hospital, which was held at the institution, and he was re-elected President, while the other officers who retired were also re-elected and thanked for their past services. The Medical Board strongly supported the Board of Management in its determination to proceed with the complete scheme of extension and the efforts to raise the £70,000 required. It was stated that there had been no occasion to close any ward owing to the outbreak of infectious disease, and this was attributed to the restrictions placed on the visits of parents to their children. The Board said the hospital could now boast of the best-equipped nurses' home in the district.

### NORTH DEVON INFIRMARY.

At the annual meeting of the Governors it was reported that the Committee had elicited that to fit up a room for dental work would cost between £25 and £30. Mr. R. P. Dunn-Pattison said the Hospital at present was very likely to run into a very heavy deficit, and he did not know whether they would be justified in spending the sum mentioned. They could not get an idea of what the current expenses would be ; they only knew the initial expenses. He thought it would be good for them to get advice from other hospitals as to what they had done, and the cost, before arriving at a definite conclusion. After discussion it was decided that the House Committee be requested to consider the question of providing a new dental department for the institution, as well as the question of finance, and report at the next meeting of the Governors.

### THE MARGARET STREET HOSPITAL FOR CONSUMPTION.

This hospital is one of those which are issuing appeals to the public this winter for money. Lord Peel and a special committee are now making an effort to raise funds for building a sanatorium which shall accommodate men, women, and children. The present Fairlight Sanatorium at Margate is altogether inadequate for the requirements of the patients whom the medical staff would like to send there, and can, moreover, admit men only. Five months has not uncommonly to elapse before a patient recommended from the out-patient department can be received there. A thousand pounds is now in hand, and it is hoped to raise another nine thousand. By Christmas the committee trust they may get a nucleus promised sufficient to enable the scheme to be put in hand and definite plans made.

### NORTHERN INFIRMARY, INVERNESS.

The new wards, which have been erected at a cost of £1,000 and carried out from admirable designs by Messrs. Alexander Ross and Sons, architects, were opened last week. They have been constructed at the rear of the main buildings. The difficulty of attaching it to the old building, and still keeping the wards isolated from the main wards, was got over by placing the additions on the ground occupied by the old operating room. Access is got to the wards by a staircase and corridor leading from the main staircase, and the wards, although attached to the old building, are completely isolated. They have a westerly exposure. The new building comprises two separate and well-lighted wards for males and females. The size of the wards is 27 feet 3 inches by 17 feet 6 inches, and the height 13 feet. At either end are placed an annexe containing bath and a lavatory, with hot and cold water. Outside the entire addition extends a verandah, 6 feet wide, supported by iron columns. The floor of the verandah is concreted, and is covered by a glass roof. The beds containing patients may be wheeled into the verandah when weather permits. Provision is made in each of the wards for five beds, and there are two single wards. A small kitchen has communication with the main kitchen, and there is a scullery, etc. The building is complete in every detail for the sanatorium treatment of the patients.

